# Syndrome de Fahr secondaire à une hypoparathyroïdie primaire: à propos d’un cas

**DOI:** 10.11604/pamj.2017.26.2.10689

**Published:** 2017-01-04

**Authors:** Fatima El Boukhrissi, Ghizlane Zoulati, Issam En-nafaa, Hassan Ouleghzal, Sara Derrou, Soumaya Safi, Youssef Bamou, Lhoussine Balouch

**Affiliations:** 1 Service de Biochimie-Toxicologie, Hôpital Militaire Moulay Ismail Meknès, Maroc; 2 Université Sidi Mohamed Ben Abdellah, Faculté de Médecine et de Pharmacie de Fès, Maroc; 3 Service de Radiologie, Hôpital Militaire Moulay Ismail Meknès, Maroc; 4 Service d’Endocrinologie, Hôpital Militaire Moulay Ismail Meknès, Maroc; 5 Université Mohamed V, Faculté de Médecine et de Pharmacie de Rabat, Maroc

**Keywords:** Fahr´s syndrome, intracerebral calcification, hyperparathyroidism, Fahr syndrome, intracerebral calcifications, hypoparathyroidism

## Abstract

Le syndrome de Fahr est une entité anatomoclinique rare dont la principale étiologie est l'hypoparathyroïdie primitive ou postopératoire. Il est caractérisé par des calcifications intracérébrales bilatérales et symétriques, localisées dans les noyaux gris centraux, le plus souvent associées à des troubles du métabolisme phosphocalcique. Les auteurs rapportent le cas d’un patient âgé de 54 ans suivi depuis 20 ans pour hypoparathyroïdie primaire, qui a présenté des troubles amnésiques révélant un syndrome de Fahr secondaire à l’hypoparathyroïdie.

## Abstract (en)

Fahr syndrome is a rare anatomo-clinic disease whose most common cause is primary or postoperative hypoparathyroidism. It is characterized by bilateral and symmetrical intracerebral calcifications located in the central gray nuclei, most often associated with phosphocalcium metabolism disorders. We here report the case of a 54-year old patient who had been treated for primary hypoparathyroidism for 20 years, presenting with amnesic disorders revealing Fahr syndrome secondary to hypoparathyroidism.

## Introduction

Le syndrome de Fahr est une entité anatomoclinique rare, caractérisée par la présence des calcifications intracérébrales bilatérales et symétriques, non artériosclérotiques, localisées dans les noyaux gris centraux [1, 2]. Cette affection est souvent associée à des troubles du métabolisme phosphocalcique, secondaire principalement à une hypoparathyroïdie primitive ou postopératoire [2]. La triade de Fahr se définit par l’association de calcifications symétriques des noyaux gris centraux, de symptômes neuropsychiatriques et d’un hypofonctionnement de la glande parathyroïde [1]. Les auteurs rapportent le cas d’un patient hospitalisé pour des troubles mnésiques et dont les examens ont permis la découverte d’un syndrome de Fahr secondaire à une hypoparathyroïdie primitive.

## Patient et observation

**Figure 1:**
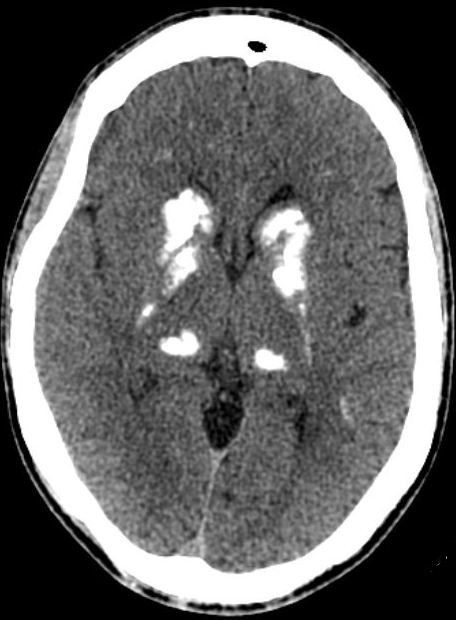
TDM cérébrale en coupe axiale sans injection du produit de contraste montrant des calcifications des noyaux caudes, noyaux lenticulaires et des thalamus

Il s’agit d’un patient âgé de 54 ans suivi depuis 20 ans pour une hypoparathyroïdie primaire. Devant la présence des troubles mnésiques et comportementaux, une tomodensitométrie (TDM) cérébrale a été réalisé, montrant des calcifications bilatérales et symétriques des deux noyaux caudés et lenticulaires (Figure 1). Le bilan phosphocalcique, le dosage de la parathormone (PTH) intacte et de la 25 hydroxy-vitamine D ont permis de mettre en évidence une hypoparathyroïdie marquée par une hypocalcémie sévère à 68,2 mg/l (VN: 85-100), une hyperphosphorémie à 57,3mg/l (VN: 27-45), une PTH basse à 2,7 pg/ml (VN: 15-65) et une 25 OH vitamine D effondrée à 9 µg/L (VN: 29-50). La numération formule sanguine, l’ionogramme sanguin, le bilan hépatique, le bilan rénal et l’hémoglobine A1c étaient normaux. Le diagnostic de Syndrome de Fahr secondaire à une hypoparathyroïdie primaire était retenu et un traitement substitutif par calcivitamine D était démarré en association avec une prise en charge neuropsychiatrique. L’évolution était marquée par la correction des troubles du métabolisme phosphocalcique et des signes neuropsychiatriques. La TDM de contrôle est restée inchangée.

## Discussion

Le syndrome de Fahr (SF) est défini par la présence de calcifications cérébrales, non artériosclérotiques, bilatérales et symétriques touchant les noyaux gris centraux. C'est une affection rare, caractérisée par un polymorphisme clinique avec une prédominance des manifestations neuropsychiatriques et des troubles du métabolisme phosphocalcique. Le SF survient préférentiellement chez les patients présentant des dysparathyroïdies, dont principalement l’hypoparathyroïdie [1–4]. Les mécanismes physiopathologiques qui concourent à la survenue des calcifications intracérébrales au cours du SF sont mal élucidés. La plupart des auteurs évoquent un trouble métabolique des cellules oligogliales avec dépôts de mucopolysaccharides et apparition secondaire de lésions vasculaires, périvasculaires et d’incrustations calcaires. Ces calcifications intéressent les petits vaisseaux des noyaux gris centraux [1–5]. Leur analyse biochimique a montré une matrice organique, constituée de mucopolysaccharides neutres et d’acides ainsi que des éléments minéraux (calcium, phosphore, fer, soufre, magnésium, aluminium, zinc) [5]. Ces calcifications se manifestent le plus souvent par des troubles neuropsychiques. L’hypoparathyroïdie est la cause la plus fréquente d’hypocalcémie liée au SF. L’hypocalcémie engendrée par l’hypoparathyroïdie explique la majorité des signes cliniques (cataracte, malabsorption, hyperexcitabilité neuromusculaire, signes neurologiques et neuropsychiques divers, désordres psychiatriques pouvant aller jusqu’à la psychose, troubles cardiovasculaires divers) [6–8]. Il est important de ne pas confondre le SF avec les autres affections pouvant engendrer des calcifications intracérébrales notamment les endocrinopathies (hypothyroïdie, hypogonadisme), les pathologies systémiques (la sclérodermie systémique, le lupus érythémateux disséminé, la maladie cœliaque) les infections (la toxoplasmose, la neurocysticercose, la rubéole), les maladies diverses (insuffisance rénale chronique, intoxication à la vitamine D, cytopathies mitochondriales) et les tumeurs cérébrales primitives ou secondaires calcifiées. Cependant, les calcifications intracérébrales observées au cours de ces différentes pathologies ont des sièges et des aspects différents [9]. En contraste avec la gravité des symptômes dont il peut être responsable, le SF est de bon pronostic et la correction des troubles du métabolisme phosphocalcique amène souvent une amélioration notable [2, 4].

## Conclusion

Le SF est une maladie neurologique rare, réalisant un contraste entre une symptomatologie sévère non spécifique et un traitement simple et efficace. La correction des troubles du métabolisme phosphocalcique permet une amélioration marquée de la symptomatologie clinique, d’ou l’intérêt de rechercher ces troubles systématiquement devant toute manifestation neuropsychiatrique associé à des calcifications des noyaux gris centraux.

## References

[1] Fahr T (1930-1931). Idiopathische Verkalkung der Hirngefässe. Zentralbl Allg Pathol..

[2] Morgante L, Trimarchi F, Benvenga S (2002). Fahr’s disease. Lancet..

[3] El Maghraoui A, Birouk N, Zaim A (1995). Fahr syndrome and dysparathyroidism. 3 cases. Presse Med..

[4] El Hechmi, Bouhlel S, Melki W, El Hechmi Z (2014). Trouble psychotique secondaire à un syndrome de Fahr: à propos d’une observation. Encephale..

[5] Finsterer J (2004). Mitochondriopathies. Eur J Neurol..

[6] Rharrabti S, Darouich I, Benbrahim M, Belahsen F, Rammou I, Alouane R (2013). Un syndrome confusionnel révélant un syndrome de Fahr avec hyperparathyroïdie. Pan Afr Med J..

[7] Chouaib N, Rafai M, Belkouch A, Bakkali H, Belyamani L (2015). Découverte fortuite d’un syndrome de Fahr suite à une crise convulsive. Rev Neurol (Paris)..

[8] Otheman Y, Khalloufi H, Benhima I, Ouanass A (2011). Manifestations neuropsychiatriques révélant une pseudohypoparathyroïdie avec un syndrome de Farh. Encéphale..

[9] Rafaia MA, Oumaria S, Lytima S, Boulaajajc FZ, El Moutawakkila B, Slassi I (2014). Le syndrome de Fahr: aspects cliniques, radiologiques et étiologiques. Feuillets de radiologie..

